# Evaluation of ultrasound-guided glossopharyngeal nerve block technique: A prospective observational study^☆^

**DOI:** 10.1016/j.inpm.2026.100744

**Published:** 2026-02-24

**Authors:** Ayushi Bansal, Sujeet Gautam, Ravisankar Manogaran, Prabhakar Mishra, Arun Kumar Gupta, Sanjay Kumar, Sandeep Khuba

**Affiliations:** a Department of Anesthesiology, Sanjay Gandhi Post Graduate Institute of Medical Sciences, Lucknow, India; b Department of Neuro-otology, Sanjay Gandhi Post Graduate Institute of Medical Sciences, Lucknow, India; c Department of Biostatistics & Health Informatics, Sanjay Gandhi Post Graduate Institute of Medical Sciences, Lucknow, India

**Keywords:** Glossopharyngeal neuralgia, Nerve block, Ultrasound guided, Image guided, Sternocleidomastoid muscle, Neck pain

## Abstract

**Background:**

Glossopharyngeal nerve block provides long-term pain relief in glossopharyngeal neuralgia patients; the nerve block can be performed using landmarks or ultrasound guidance. The present study has evaluated the efficacy of ultrasound-guided glossopharyngeal nerve block utilizing the small-sized hockey stick probe.

**Methods:**

The present study was a prospective, observational study; twenty-five adult patients diagnosed with primary glossopharyngeal neuralgia not responding to medical management were included in this clinical trial. Glossopharyngeal nerve block was done under ultrasound guidance using hockey stick probe; patients having more than 50% reduction in numeric rating scale (NRS) score, for at least 2 h following nerve block, were enrolled in the study and followed for 6 months. The primary outcome measure was the severity of pain, measured by NRS score. Secondary outcome measures were percentage pain relief, reduction of analgesic usage, and PHQ-9 score for psychological assessment. All these assessments were done prior to the procedure and at 2 weeks, 1, 3 and 6 months after the procedure.

**Results:**

We observed significant reduction in the NRS scores at 2 weeks (1.7 ± 1.6), 1 (1.9 ± 1.3), 3 (1.8 ± 1.3) and 6 months (2.1 ± 1.5) after Glossopharyngeal nerve block as compared to the baseline (6.1 ± 1.3; P value < 0.05); we also observed a significant pain relief (76%) and significantly reduced analgesic consumption (68%) and PHQ-9 scores (2.3 ± 1.7) compared to the baseline values (P value < 0.05).

**Conclusion:**

Ultrasound-guided glossopharyngeal nerve block with a linear array hockey stick probe provided significant pain relief in 75% of study participants with glossopharyngeal neuralgia over a six-month follow-up period.

## 1 Introduction

Glossopharyngeal neuralgia is characterized by sudden, intense episodes of sharp, stabbing pain affecting areas supplied by the glossopharyngeal nerve, such as the ear, base of the tongue, throat, tonsillar region, and angle of the jaw [1]. These painful episodes are often triggered or worsened by activities such as swallowing, coughing, or speaking, which can significantly impact a person's quality of life. While most cases are idiopathic, potential underlying causes include an elongated styloid process (Eagle syndrome), complications after tonsillectomy, skull base tumors, vascular compression, and neurological conditions such as multiple sclerosis [1,2].

Management of glossopharyngeal neuralgia includes medical therapy, nerve blocks, pulsed radiofrequency treatment, and surgical interventions [1,2]. First-line treatment typically involves carbamazepine or oxcarbazepine. For patients who do not experience adequate relief with medications, a glossopharyngeal nerve block followed by pulsed radiofrequency treatment may be used. Surgical options, such as microvascular decompression or rhizotomy, are considered for those who remain resistant to conservative treatments. Glossopharyngeal nerve block provides long-term pain relief with minimal side effects. It can be performed using landmark-based techniques or under ultrasound, fluoroscopic, or CT guidance [[3], [4], [5]].

**Fig. 1:**
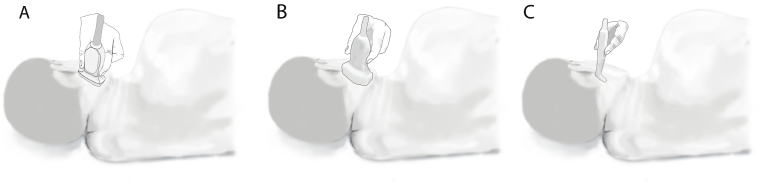
Ultrasound guided Glossopharyngeal Nerve Block with Linear (A), Curvilinear (B) and Hockey stick (C) ultrasound probe placed between mastoid process and angle of mandible.

Ultrasound-guided glossopharyngeal nerve block offers the benefit of real-time visualization of important anatomical structures along with direct monitoring of injectate spread. This improves procedural accuracy and lowers the risk of complications, especially inadvertent intravascular injection. Although the technique is often performed using linear or curvilinear ultrasound probes [3,4], their larger size can make the ultrasound probe handling difficult in the narrow space between mastoid process and mandibular angle (Fig. 1). To overcome this, we recommend using a compact hockey stick probe, which provides better access and easier needle guidance in this anatomically constrained region. In this study, we evaluated the efficacy of ultrasound-guided glossopharyngeal nerve block using a small-sized hockey stick probe.

## 2 Material and methods

### 2.1 Study Design

The present study was a prospective, observational study conducted after approval from the “Exp-54th Institute's ethics committee” (IEC code: 2023-256-MD-EXP-54) and obtaining written informed consent from the patients. This clinical trial has been registered in the Clinical Trials Registry, India (Registration number: CTRI/2024/02/062486).

### 2.2 Inclusion criteria

Patients over 18 years of age diagnosed with primary glossopharyngeal neuralgia that did not respond to medical management were included in this study.

### 2.3 Exclusion criteria

Patients with cardiopulmonary diseases such as myocardial infarction and heart failure; patients with mental disorders, local infections, or pregnancy; patients with abnormal coagulation function; and patients with a history of snoring.

### 2.4 Study intervention

**Fig. 2:**
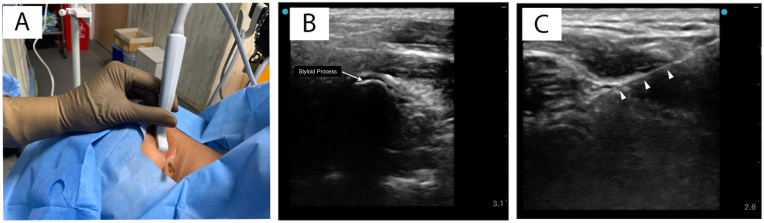
Ultrasound guided Glossopharyngeal Nerve Block. A. Hockey stick probe placement B. Ultrasound image demonstrating the styloid process and the acoustic shadow C. Ultrasound image demonstrating the Needle track (solid arrows).

Patients enrolled in this study underwent ultrasound-guided glossopharyngeal nerve block. The procedure was performed under standard hemodynamic monitoring with the patient lying in the lateral position, supported by a thin pillow under the head. Using aseptic precautions, the area between the mastoid process and the angle of the mandible was scanned with a high-frequency (18-6 MHz) linear array hockey stick probe (GE LOGIQ E) to locate the styloid process. Color flow Doppler was then used to identify the internal carotid artery and the internal jugular vein adjacent to the styloid process. A 22-gauge, 3.5-inch needle was inserted using an in-plane approach directed toward the styloid process; the styloid process was contacted, and the needle was advanced posteriorly (Fig. 2). Subsequently, 2 ml of 1% lidocaine with 4 mg of dexamethasone was injected under real-time ultrasound guidance after confirming negative aspiration for blood. A positive response was defined as a reduction of greater than 50% in numerical rating scale (NRS) scores for at least 2 h following lidocaine injection. Patients exhibiting a positive response were followed for a period of 6 months.

Post-procedure patients were monitored for 2 h before being discharged. A fixed-dose analgesic combination of acetaminophen (325 mg) and tramadol (37.5 mg) was prescribed three times daily for five days following the procedure. Afterward, the patient was advised to take the same analgesic as needed if the NRS score exceeded 3 during the follow-up period. A repeat glossopharyngeal nerve block was performed whenever pain severity increased to an NRS score of over 5 or pain relief decreased to less than 50% [6].

### 2.5 Outcome measures and assessment

The primary outcome measure was pain severity, with a reduction of 50% or more considered significant. This was assessed using the NRS score, which is an 11-point scale from 0 to 10, where 0 indicates no pain and 10 indicates the worst imaginable pain.

Secondary outcome measures included percentage pain relief (with ≥50% pain relief considered significant), reduction in analgesic use, and psychological assessment to detect severity of depression was done using the PHQ-9 questionnaire (Diagnostic and Statistical Manual of Mental Disorders, 4th edition, 1994; PHQ-9: Patient health questionnaire with 9 items) [7]. A reduction of 50% or more in the analgesic dose was regarded as a decrease in analgesic use. The daily analgesic requirement of each patient was recorded before the procedure. All assessments were conducted at baseline (before the procedure), 2 h after the procedure, and at 2 weeks, 1 month, 3 months, and 6 months post-procedure. Follow-up assessments between 2 weeks and 6 months were performed via telephone [6].

### 2.6 Sample size estimation and statistical analysis

Using a one-tailed Wilcoxon signed-rank test for matched pairs (as a reduction in score was anticipated), with an assumed effect size of 1, a significance level (α) of 0.05, and a power of 0.95, the required sample size was estimated to be 15. The effect size was derived from an expected absolute reduction in pain score of 3 with a standard deviation of 3. After adjusting for a design effect of 1.5 and an anticipated 10% loss to follow-up, the final sample size was increased to 25; accordingly, 25 patients were enrolled in the study.

Continuous variables are presented as mean ± standard deviation. The Wilcoxon signed-rank test was used to compare the NRS and PHQ-9 scores between pre- and post-treatment. Categorical variables are presented as numbers (percentages). The McNemar test was applied to compare pre- and post-treatment outcomes for percentage pain relief and reduction in analgesic dose. A P value < 0.05 was considered statistically significant. Data analysis was performed using the Statistical Package for the Social Sciences, version 26 (SPSS v26; IBM Corp., Chicago, IL, USA).

## 3 Results

**Fig. 3:**
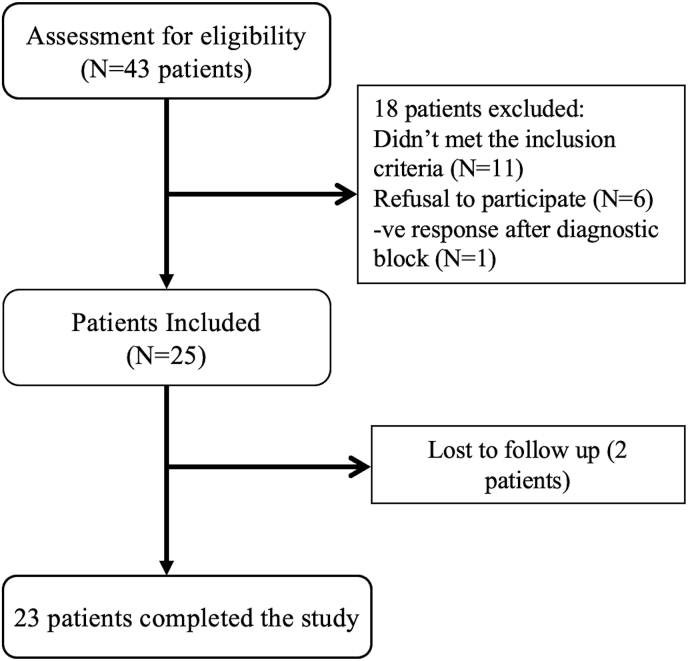
Study design.

The study was conducted between February 2024 and March 2025. A total of 43 patients with glossopharyngeal neuralgia were interviewed (Fig. 3), of whom 25 were enrolled. Of the remaining 18 patients, 11 did not meet the inclusion criteria, 6 declined participation, and 1 patient did not respond positively to the diagnostic block. Twenty-three patients completed the trial; two patients were lost to follow-up—one after two months and the other after four months of follow-up; for these individuals the last recorded observation was carried forward. Demographic details are provided in Table 1.

**Table 1:** Patient demographics.

Demographic Variable	Demographic Data (N = 25)
Age (Years)	38.3 ± 11.4
Gender (Male: Female)	7:18
Weight (Kg)	56.3 ± 8.5
Median Duration of Pain [months (IQR)]	24 (12)
Mean Baseline analgesic use (Paracetamol 325 mg + Tramadol 37.5 mg combination)	Paracetamol: 910 ± 162.5 mg + Tramadol 103.5 ± 16.3 mg

Data are presented as either mean values ± SD, median (interquartile range) or by absolute numbers.

All patients in the study experienced submandibular pain. Additionally, 11 patients (44%) reported throat pain, 5 patients (20%) reported ear pain, 7 patients (28%) reported retro-auricular pain, and 3 patients (12%) reported neck pain. Pain was worsened by swallowing in 6 patients (24%) and during talking in 2 patients (8%).

Ultrasound-guided glossopharyngeal nerve block led to a significant decrease in NRS pain scores at 2 weeks, and at 1, 3, and 6 months after the procedure compared to baseline values (Table 2; P < 0.05). Three patients needed a repeat glossopharyngeal nerve block because their NRS scores exceeded 5 at the 3-month (2 patients) and 4-month follow-up (1 patient). PHQ-9 scores were significantly reduced at all follow-up intervals except at 2 weeks (Table 3; P < 0.05); most patients experienced significant pain relief [17 patients (68%) at 6 months] and lower analgesic use [19 patients (76%) at 6 months] during the 6-month follow-up period (Table 3). All patients enrolled in the trial were using paracetamol-tramadol combination as analgesic at baseline; the median daily analgesic consumption was reduced from three tablets (prior to procedure) to zero tablet (at 2 weeks and 1 month) and one tablet (at 3 and 6 months) of paracetamol-tramadol combination; the mean morphine milligram equivalent (MME) for tramadol in analgesic use decreased from a baseline of 20.7 ± 3.3 mg to 1.5 ± 3.1 mg at 2 weeks, 3.3 ± 6.2 mg at 1 month, 6.0 ± 5.7 mg at 3 months, and 8.1 ± 6.8 mg at 6 months.

**Table 2:** Outcome measures (pain scores).

Follow up period	NRS Score (N = 25)
Baseline	6.1 ± 1.3
2 weeks	1.7 ± 1.6 * (P = 0.001)
1 month	1.9 ± 1.3 * (P = 0.001)
3 months	1.8 ± 1.3 * (P = 0.001)
6 months	2.1 ± 1.5 * (P = 0.001)

Data are presented as mean values ± SD, absolute numbers; NRS: Numerical Rating Scale. Wilcoxon signed-rank test used to compare the baseline values with other follow up periods. P < 0.05 significant.

**Table 3:** Outcome measures.

Follow up period	Patients with significant percentage pain relief ** (N = 25)	Patients with reduced analgesic consumption a (N = 25)	PHQ-9 Score (N = 25)
Baseline	NA (95% CI [0-13.7%])	N/A (95% CI [0-13.7%])	5.2 ± 3.3
2 weeks	25 * (100%)	25 * (100%)	5.2 ± 3.3 (P > 0.05)
	(95% CI [86.3-100%])	(95% CI [86.3%-100%])	
	(P = 0.001)	(P = 0.001)	
1 month	22 * (88%)	20 * (80%)	2.4 ± 2.7 * (P = 0.001)
	(95% CI [68.8-97.5%])	(95% CI [59.3-93.2%])	
	(P = 0.001)	(P = 0.001)	
3 months	20 * (80%)	20 * (80%)	2.1 ± 2.2 * (P = 0.001)
	(95% CI [59.3-93.2%])	(95% CI [59.3-93.2%])	
	(P = 0.001)	(P = 0.001)	
6 months	19 * (76%)	17 * (68%)	2.3 ± 1.7 * (P = 0.001)
	(95% CI [54.9-90.6%])	(95% CI [46.5-85.1%])	
	(P = 0.001)	(P = 0.001)	

*Data are presented as mean values* ± SD, absolute numbers or 95% confidence intervals (CI); PHQ-9: Patient health questionnaire with 9 items; *P<0.05 during comparison of baseline values with other follow up periods; ** significant percentage pain relief was ≥50% pain relief.

a reduced analgesic consumption was a reduction of 50% or more in the analgesic dose.

## 4 Discussion

The current study demonstrated that an ultrasound-guided glossopharyngeal nerve block using a linear array hockey stick probe effectively decreased pain and analgesic needs in patients with primary glossopharyngeal neuralgia.

The ultrasound-guided glossopharyngeal nerve block is performed by positioning the ultrasound probe within the narrow space between the angle of mandible and the mastoid process. While linear and curvilinear probes typically have a footprint width of 4.2 to 6.6 cm, the hockey stick probe used in this study has a smaller footprint width of approximately 2.6 cm (LOGIQ E; GE Healthcare; Wauwatosa, Wisconsin). Compared to the larger linear and curvilinear probes, the smaller size of the hockey stick probe makes it easier to handle in this anatomically constrained area. However, its reduced footprint can make it more difficult to visualize underlying structures over a larger area. In this case, the target area for the glossopharyngeal nerve block was superficial and confined between the narrow anatomical margins of the angle of mandible and mastoid process, which allowed for clear identification of relevant structures despite the smaller probe size.

In the present study, ultrasound-guided glossopharyngeal nerve block with a hockey stick probe provided significant pain relief and lowered analgesic needs in over 80% of cases at one month, with sustained benefits seen in 70% of patients at six-month follow-up. Many of these individuals had long-standing fears that their pain was due to a malignant condition; the effective relief achieved through this minimally invasive procedure reassured them that their condition was both benign and manageable. At the six-month follow-up, four patients reported mild submandibular pain with tenderness over the sternocleidomastoid muscle, which responded well to sternocleidomastoid stretching exercises. Our observations suggest that sternocleidomastoid muscle spasm may coexist with glossopharyngeal neuralgia, and a thorough clinical history and physical examination are key to identifying and managing this component effectively.

The efficacy of ultrasound-guided glossopharyngeal nerve block reported in retrospective studies by Liu et al. [3] was 83% at six months and 77% at one year by You et al. [4]; both of these were retrospective studies, and treatment efficacy was defined by a reduction of 2 points in the visual analogue scale score by Liu et al. or by achieving Barrow neurological institute scale I after treatment. The reported efficacy by Liu et al. [3] and You et al. [4] was higher as compared to the present study. However, differences in the parameters used to assess efficacy make comparing the results of the present study with these retrospective studies unfeasible. We could not find any prospective study in the literature evaluating the efficacy of ultrasound-guided glossopharyngeal nerve block.

In the present study, three patients (12%) required a repeat glossopharyngeal nerve block; modalities like pulsed radiofrequency treatment of the glossopharyngeal nerve [5,8,9] may be used to provide long-term pain relief in cases where pain recurs. Pulsed radiofrequency treatment offers sustained analgesia by neuro-modulatory changes in the nerve through the strong magnetic field generated around it [10]. The use of less invasive, ultrasound-guided glossopharyngeal nerve block helps to minimize the need for more invasive procedures like microvascular decompression or pulsed radiofrequency treatment.

Ultrasound-guided nerve block technique to block the sensory innervation of the nerve has gained popularity recently because of the ability to visualize bony structures, surrounding vascular structures and the real-time passage of needle [4,5]. The ability to see the drug spread has helped reduce the dose of anesthetic and the overall incidence of side effects using this technique. At the level of styloid process, cranial nerves IX, X, and XI are sufficiently separated from each other, with the glossopharyngeal nerve being the closest to the distal part of the styloid process. This approach minimizes neurovascular complications. The technique does not require the use of any contrast agent (making it safe for people with iodinated contrast allergy) and involves no radiation exposure. Additional advantages of ultrasound-guided glossopharyngeal nerve block include the improved ability to visualize the styloid process compared to fluoroscopy, as well as eliminating the need for true lateral imaging, which can often present technical challenges.

## 5 Study limitations

Firstly, the sample size is too small to comment on the outcome measures, including percentage pain relief, DSM-IV, and reduction in analgesic consumption. A clinical trial with a control group and a larger sample size is needed to draw stronger conclusions about these outcome measures. Secondly, a follow-up period of 6 months is too short to adequately assess the above outcomes. Thirdly, needle placement and positioning may be challenging in individuals with short necks. Clinicians might find it difficult to locate the styloid process using ultrasound, which is associated with a longer learning curve and interobserver variability. Additionally, this study was conducted at a single center, so a multi-center study is necessary to obtain higher-level evidence.

## 6 Conclusion

In this study, ultrasound-guided glossopharyngeal nerve block provided significant pain relief in 75% of participants with glossopharyngeal neuralgia over a six-month follow-up. The results also show that the procedure can be effectively performed using a compact linear array hockey stick probe, which improves manoeuvrability.

## Source(s) of financial support

Nil.

## Presentation at a meeting

Nil.

## Ethical approval and informed consent

The present study was a prospective, observational study conducted after approval from the Institute's ethics committee (IEC code: 2023-256-MD-EXP-54) and obtaining written informed consent from the patients. This clinical trial has been registered in the Clinical Trials Registry, India (Registration number: CTRI/2024/02/062486).

## Funding sources

None.

## Conflicting interest

Nil.
